# Novel *ABCA4* compound heterozygous mutations cause severe progressive autosomal recessive cone-rod dystrophy presenting as Stargardt disease

**Published:** 2009-04-03

**Authors:** Quansheng Xi, Lin Li, Elias I. Traboulsi, Qing Kenneth Wang

**Affiliations:** 1 Center for Cardiovascular Genetics and Department of Molecular Cardiology, Lerner Research Institute, The Cleveland Clinic, Cleveland, OH; 2 Department of Molecular Medicine, Cleveland Clinic Lerner College of Medicine of Case Western Reserve University, Cleveland, OH; 3 Key Laboratory of Molecular Biophysics of the Ministry of Education, College of Life Science and Technology and Center for Human Genome Research, Huazhong University of Science and Technology, Wuhan, People’s Republic of China; 4 Cole Eye Institute, The Cleveland Clinic, Cleveland, OH

## Abstract

**Purpose:**

To identify the gene causing a severe form of progressive autosomal recessive cone-rod dystrophy presenting as Stargardt disease and to characterize clinical features in a large American family.

**Methods:**

We characterized an American family who had an unusual retinal dystrophy with clinical features of Stargardt disease and severe progressive cone-rod dystrophy. Family members underwent complete ocular examinations with evaluation of visual acuity, visual fields, fundus examination, fluorescein angiography, and electroretinography. Genome-wide linkage analysis of the family was performed using 408 microsatellite markers spanning the entire human genome. Direct DNA sequence analysis was used for mutational analysis of the *ABCA4* gene in all exons and exon-intron boundary regions and for testing cosegregation of the mutations with the disease in the family. DNA sequence analysis was used to determine the presence of the mutations in 200 unrelated controls.

**Results:**

The proband presented with a clinical phenotype that was initially compatible with Stargardt disease, only to progress to a severe cone-rod dystrophy over the course of a few years. The disease-causing gene in the family was linked to the *ABCA4* locus on chromosomal 1p22. One novel mutation, c.655A>T, was identified in exon 6 and another novel splicing mutation, c.5312+3A>T, was identified in intron 37 of *ABCA4*. The mutations were not present in 200 controls. The two affected sisters in this pedigree were compound heterozygotes for the mutations. Unaffected family members either did not carry either or had only one of the two mutations.

**Conclusions:**

We have identified two novel *ABCA4* mutations, c.655A>T and c.5312+3A>T. When present as a compound heterozygous state, the mutations cause a phenotype of retinal dystrophy that initially manifests as Stargardt disease and slowly progresses to a severe cone-rod dystrophy. These results expand the wide range of clinical manifestations of *ABCA4* mutations.

## Introduction

Stargardt disease is the most common type of hereditary macular dystrophy. It is characterized by decreased central vision, atrophy of the macula and underlying retinal pigment epithelium, and the frequent presence of prominent flecks in the posterior pole of the retina. To date, mutations in three genes have been identified as causing Stargardt disease, including ATP-binding cassette, subfamily A, member 4 (*ABCA4*), elongation of very long chain fatty acids-like 4 (*ELOVL4*), and cyclic nucleotide-gated channel, beta-3 (*CNGB3*) [1-3]. A fourth locus for Stargardt disease was mapped to chromosome 4p [4], but the specific gene has not been identified yet.

The *ABCA4* gene encodes a retinal-specific ATP-binding cassette transporter (ABCR) with 2,273 amino acid residues and a molecular weight of 220 kDa [5]. The ABCR protein is mainly expressed in the outer segment disks of rods and cones and involved in the transport of retinoid derivatives across the outer segment disk membrane [6-8]. ABCR may also act in the visual cycle to flip retinal pigment epithelium all-trans-retinal adducts from the lumenal to the cytosolic face of the disk membrane, move free all-trans-retinal from the lipid phase of the disk membrane to a juxtamembrane location, or possibly reorient all-trans-retinal in the bilayer [9,10].

Mutations in *ABCA4* are responsible for almost all cases of classic Stargardt disease. *ABCA4* mutations have also been identified in fundus flavimaculatus, cone-rod dystrophy, retinitis pigmentosa, and age-related macular degeneration [11-15]. To explain the wide spectrum of vision disorders caused by mutations in *ABCA4*, Maugeri et al. proposed that mutant alleles with severe consequences (null mutations) cause visual disorders with severe clinical features, while mild mutations lead to mild clinical phenotypes [16].

We characterized a family with an interesting phenotype of retinal dystrophy that started as classic juvenile Stargardt disease, but rapidly progressed to a severe cone-rod dystrophy. Linkage analysis mapped the disease-causing gene to chromosome 1p22 where *ABCA4* is located. Sequence analysis of all 50 exons and exon-intron boundaries in the *ABCA4* gene identified two novel compound heterozygous mutations: one nonsense mutation and one splicing site mutation. The mutations cosegregated with affected members in the family and were not present in 200 normal controls.

## Methods

### Subjects

A 12 member Caucasian family with Stargardt disease was identified in Ohio and characterized at the Cole Eye Institute. Controls for this study were all Caucasian individuals with an average age range of 53.5±12.1 years and without apparent ophthalmic problems by physical examinations. Each study participant donated blood samples (5–20 ml), which were used for isolation of genomic DNA using the DNA Isolation Kit for Mammalian Blood (Roche Diagnostic Co., Indianapolis, IN). This study was approved by the Institutional Review Board on Human Subjects of the Cleveland Clinic, and informed consent was obtained from every participating family member.

### Molecular studies

Genome-wide genotyping was performed by the genotyping service at Marshfield Clinic Center for Medical Genetics (Marshfield, WI) with 408 microsatellite markers spanning the entire human genome by every 10 cM. For fine mapping, six new markers were selected from the University of California Santa Cruz Genome Browser database: *D1S1588, D1S1170, D1S406, D1S1174, D1S2651*, and *D1S2726*. Genotyping at these markers were performed using one fluorescence-labeled (*D1S406*) marker and five ^32^P-labeled polymorphic markers. For the fluorescence-labeled marker, polymerase chain reaction (PCR) was performed in an ABI 9700 PCR system (Applied Biosystems, Foster City, CA) in a volume of 10 ul containing PCR buffer, dNTPs, PCR primers, human genomic DNA, and Taq polymerase. PCR conditions were: denaturing at 95 °C for 12 min followed by 10 cycles of 95 °C for 15 s, 55 °C for 15 s and 72 °C for 30 s, then 20 cycles of 89 °C for 15 s, 55 °C for 15 s and 72 °C for 30 s followed by 10 min at 72 °C. After PCR, 100 µl water was added into each reaction well of PCR product and 2 µl was drawn out into a new well. Next, a mix of 12 µl formamide and 0.5 µl ROX size standards was added to each well. A ROX size standard (Applied Biosystems) is a dye-labeled size standard for the reproducible sizing analysis for fragments between 35 and 500 bp. The standard contains 16 dye-labeled, single-stranded DNA fragments. The samples were then analyzed on an ABI 3100 genetic analyzer (Applied Biosystems). Results were analyzed with GeneMapper software (version 1.1, Applied Biosystems).

For ^32^P-labeled markers, PCR was carried in a 10 ul reaction containing buffer, dNTP mix, ^32^P-labeled marker primers, Taq polymerase, and human genomic DNA. This mixture was placed in a thermocycler for 5 min of denaturing at 95 °C followed by 30 cycles of 95 °C for 30 s, 55 °C for 30 s, and 72 °C for 30 s and a final extension step at 72 °C for 10 min. PCR products were separated on 6% denaturing polyacrylamide gels, and alleles were called manually.

Mutational analysis was performed by direct DNA sequence analysis. Each exon and flanking exon-intron sequences were PCR-amplified using the standard conditions with specific primers (Appendix 1). PCR products were purified using Qiaquick gel extraction kit (Qiagen, Valencia, CA) and sequenced. Sequencing reactions were carried using Big-Dye terminator v1.1 cycle sequencing kit (Applied Biosystems) and analyzed on Genetic Analyzer 3100 (Applied Biosystems). The splicing mutation was confirmed by subcloning PCR products from the proband into the pcDNA3.1-TOPO vector (Invitrogen, Carlsbad, CA). After transformation into *E. coli* DH5α, eight colonies were picked, and the inserts were PCR amplified and sequenced. Direct DNA sequence analysis of exons where mutations were identified was used to determine whether the mutations were present in 200 controls.

## Results

The proband (II-1) was an eight and a half year-old girl who presented with progressive reduction of vision of several months duration. She had eight siblings who had no ocular problems except for her youngest sister (II-10), who had a severe form of Goldenhar syndrome with unilateral microphthalmia. The proband’s visual acuity was 20/200 in both eyes with a very mild hypermetropic refractive error. She had no strabismus and had normal stereopsis. The slit-lamp examination was normal in both eyes. Ophthalmoscopy showed subtle macular mottling of the retinal pigment epithelium without any evident flecks. The fundus periphery was normal. A fluorescein angiogram was obtained, which showed a dark choroid sign. There was hyperfluorescence around the macular area with pinpoint areas of hyperfluorescence in the posterior pole and in the midperiphery. A clinical diagnosis of Stargardt disease was made. One year later, visual acuity had decreased to 12/200, and macular pigmentary changes were evident. Individual II-4, the proband’s younger sister by three years, presented with vision loss at about the same age as the proband and was also diagnosed with Stargardt disease.

**Figure 1:**
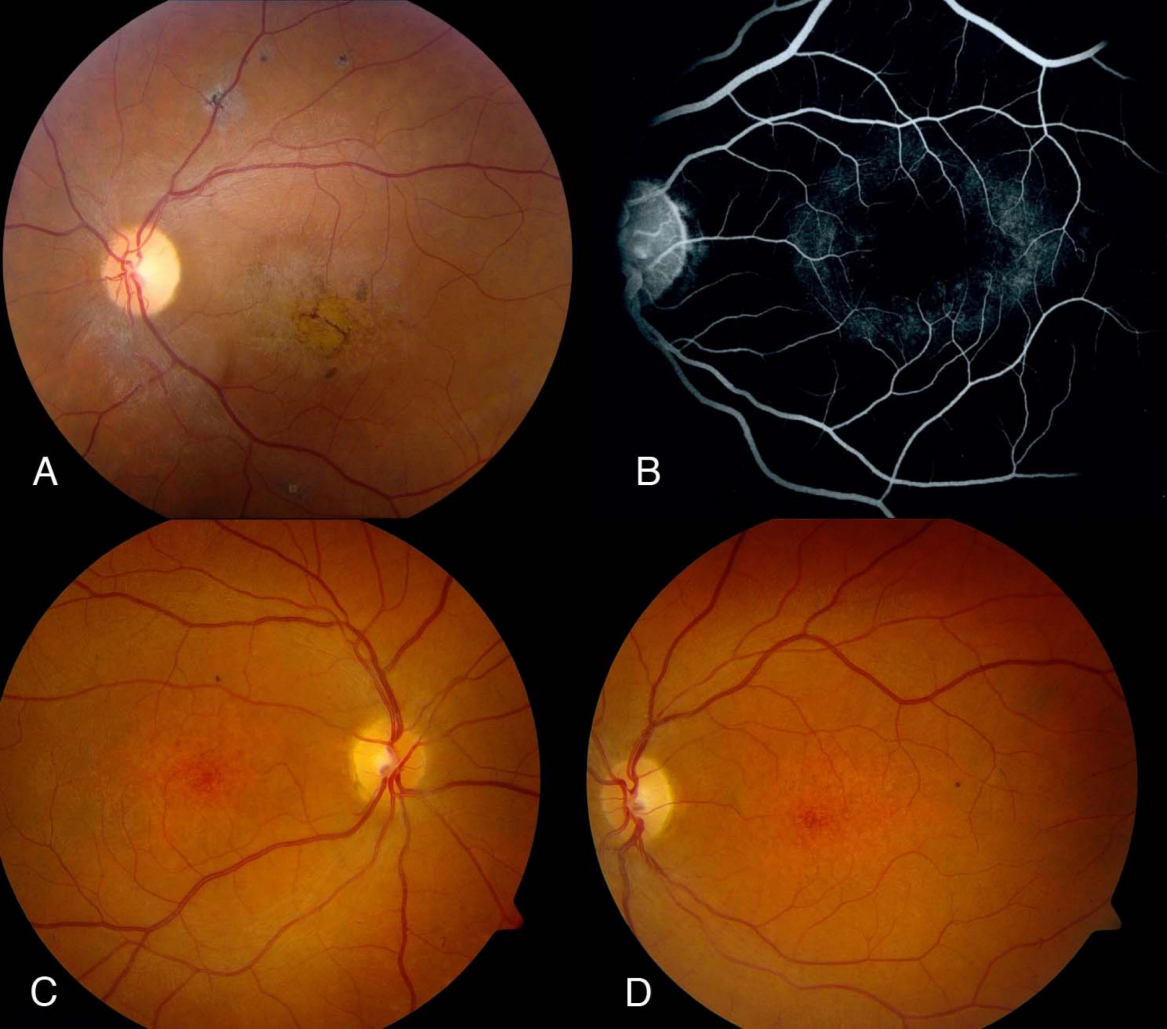
Typical retinal phenotype in affected members **A:** Left fundus of proband showing temporal optic nerve pallor, attenuated retinal blood vessels, macular pigmentary and atrophic changes, and some fine pigment clumping outside the vascular arcade. **B:** Mid-transit fluorescein angiogram of left fundus of younger sister reveals a dark choroid sign with some retinal pigment epithelium (RPE) transmission defects in a perifoveal distribution. **C **and **D:** Right and left fundus photographs of younger sister at age 12 years show mottling of RPE in macular region but no flagrant pigmentary changes; there is slight temporal pallor of the optic nerve heads, especially in left eye.

The proband’s vision continued to deteriorate slowly, and she developed temporal pallor of her optic nerve head as well as peripheral pigmentary changes in a bony spicule pattern (Figure 1A). She also developed a fine nystagmus. Her macular areas took on a beaten bronze appearance. At the age of 15 she complained of significant difficulties with dark adaptation. An electroretinogram showed no recordable waveforms under scotopic and photopic conditions. At the age of 17 her visual acuity was 20/500 OD and 4/200 OS. The peripheral pigmentary changes had become extensive, and her visual fields showed severe peripheral constriction.

Family member II-4 had a similar clinical course with minor differences. She had a similar level of visual acuity. There was a dark choroid sign on angiography (Figure 1B). Her photopic and scotopic electroretinographic responses were recordable but reduced by about 50% in amplitude. When she was last examined at the age of 14 years, she had not developed peripheral pigmentary changes, but rather depigmented spots she had in the fundus midperiphery. Her macular areas were atrophic (Figure 1C,D). Her last visual acuity was 20/450 OD and 2/200 OS.

**Figure 2:**
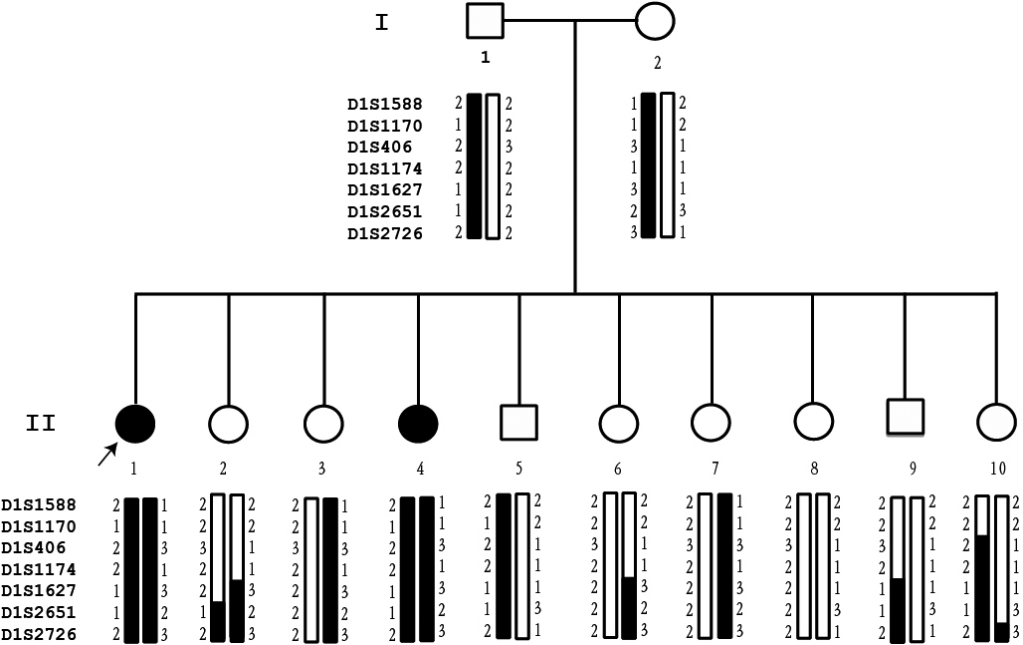
Linkage of the Stargardt family to *ABCA4*. The pedigree structure of a family with Stargardt disease and results from linkage analysis and fine mapping to the *ABCA4* locus are shown. Circles denote females, and squares indicate males. Dark symbols represent affected individuals with Stargardt disease. Empty symbols are normal individuals. The proband is marked by an arrow. The gray symbol indicates occurrence of Goldenhar syndrome but not Stargardt disease. Genotyping results for markers *D1S1588, D1S1170, D1S406, D1S1174, D1S1627, D1S2651*, and *D1S2726* are shown under each symbol. Haplotypes were constructed on the basis of the minimum number of recombinations between markers. The disease haplotype shared by all affected individuals is denoted by the blackened vertical bar, and normal haplotype is denoted by an empty vertical bar. The pedigree information is as in this figure. The proband is marked by an arrow.

The inheritance pattern in the family appeared to be autosomal recessive (Figure 2). The parents had normal eye examinations and were not consanguineous. Due to the moderate size of the family, we performed a genome-wide genotyping scan to identify the chromosomal location of the disease-causing gene in the family. A total of 408 microsatellite markers that covered the entire human genome by every 10 cM were analyzed. A positive linkage was identified with marker *D1S1627 *on chromosome 1 (position, 106,765,188 bp). Six additional markers flanking *D1S1627* were selected for fine mapping. As shown in Figure 2, haplotype analysis showed that the two affected individuals (II-1 and II-4) carried haplotype 2–1–2–2–1–1–2 from the father and haplotype 1–1–3–1–3–2–3 from the mother. None of the other siblings carried both haplotypes. Analysis of recombinant events defined the location of the disease gene above *D1S2651*.

**Figure 3:**
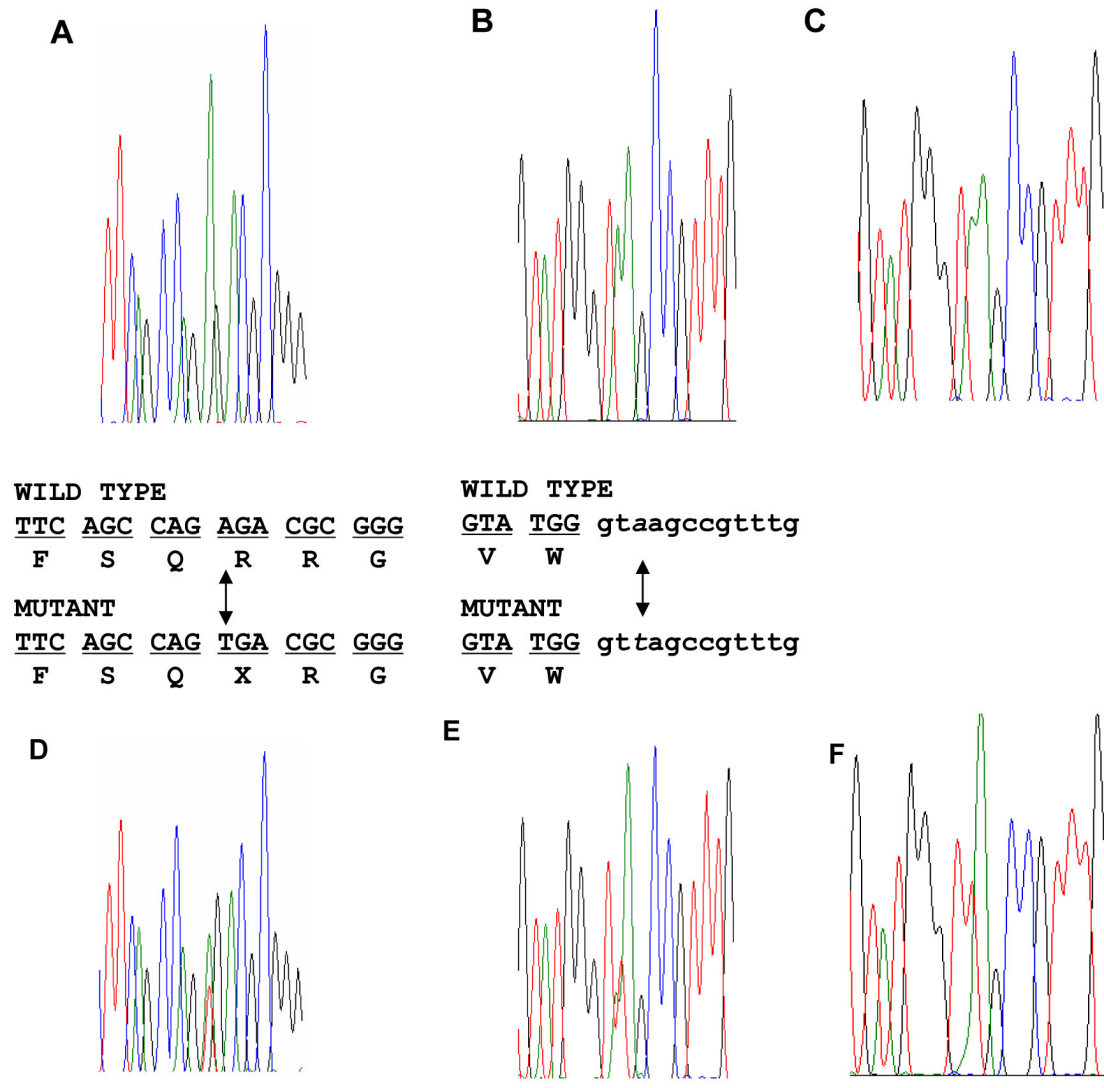
Identification of two novel *ABCA4* mutations. DNA sequence analysis for patient II-1 showed the presence of compound heterozygous c.655A>T and c.5312+3A>T mutations. **A** and **D **show the sequences from a normal and affected family member with mutation c.655A>T allele, respectively. **B** and **E** show the sequences from a normal and affected family member with mutation c.5312+3A>T allele, respectively. **C** and **F** show sequences for the wild type and mutant c.5312+3A>T allele, respectively, which were separated by subcloning of PCR products from an affected family member.

The *ABCA4* gene (position, 94,239,702–94,249,073 bp) is located within the locus and became a strong candidate gene responsible for the phenotype in the family. PCR primers were designed to amplify and sequence all exons and exon-intron boundaries of the *ABCA4* gene. Two novel mutations were identified (Figure 3). An A to T change in exon 6 (c.655A>T) resulted in a stop codon and thus generated a mutant ABCR protein (p.R219X; Figure 3A for wild type and Figure 3D for mutant sequence). The other mutation of A to T change (c.5312+3A>T) was identified at a 5′ splicing site in intron 37 (Figure 3B for wild type and Figure 3E for mutant sequence). For confirmation of the splicing mutation, PCR products were cloned and multiple clones were sequenced. Mutation c.5312+3A>T was confirmed (Figure 3C showing wild type and Figure 3F showing mutant, both from cloned PCR products). The substitution of A with T will abolish the splicing site as demonstrated by analysis with NetGene2. The mutation was predicted to introduce intron 37 into the mature ABCA4 mRNA during post-transcriptional splicing and cause a frameshift. The predicted mutant protein would have 1–1770 amino acid residues of ABCR followed by seven exogenous amino acid residues (LAVWAIS) that are translated from the sequence of intron 37. Amino acid residues 1771–2273 of ABCR would be deleted in the mutant protein.

**Figure 4:**
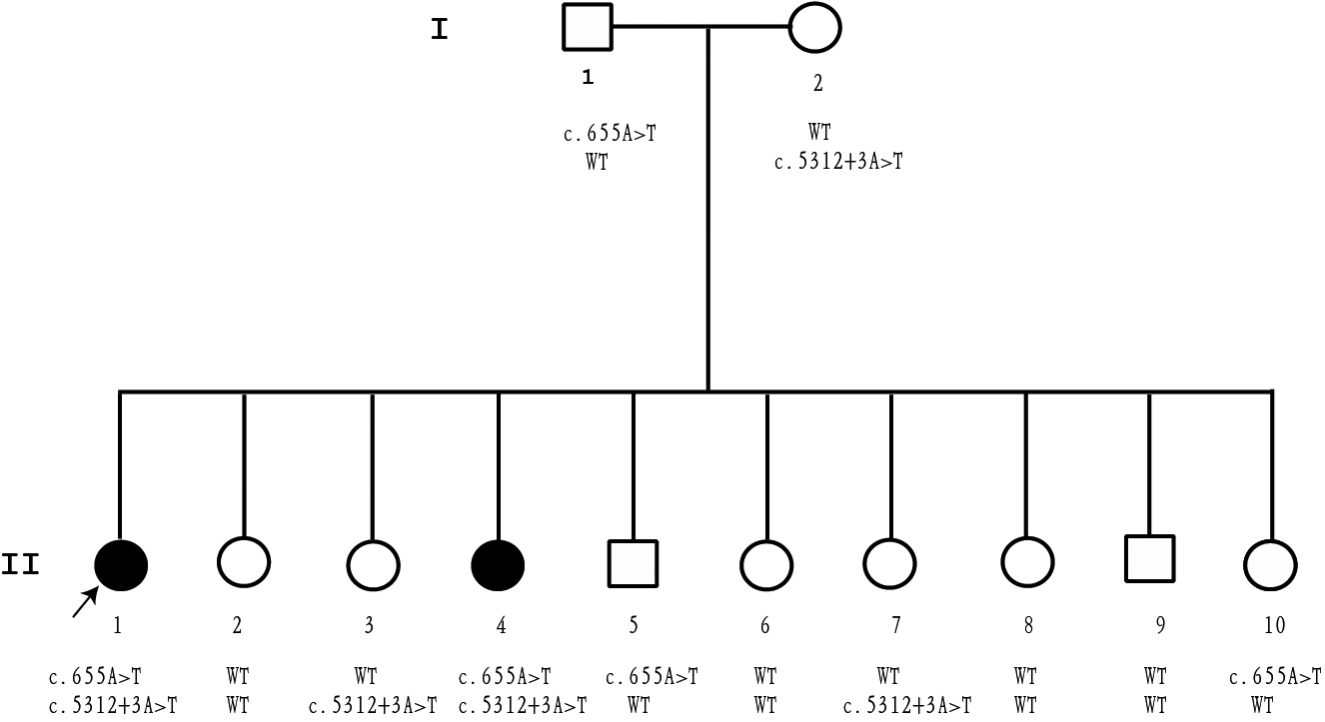
Cosegregation of two novel *ABCA4* mutations with the disease in the family. Sequence analysis on mutations c.655A>T and c.5312+3A>T were done on every family member. Both mutations cosegregated with the disease within the family. Both affected sisters inherited the c.655A>T mutant allele from their father and the c.5312+3A>T mutant allele from their mother. Normal siblings either carried both widetype alleles (II-2, 6, 8, 9) or only one mutant allele (II-3, 5, 7, 10).

Direct DNA sequence analysis was used to determine whether the mutations cosegregate with the disease in the family. As shown in Figure 4, only the two affected siblings carried both mutations, c.655A>T from the father and c.5312+3A>T from the mother. Thus, the affected individuals are compound heterozygotes for both mutations. However, the normal family members carried only one or neither of the two mutations. These two mutations were not found in 200 unrelated controls by direct DNA sequence analysis.

## Discussion

In this study, we identified two novel compound heterozygous mutations, c.655A>T and c.5312+3A>T, in the *ABCA4* gene that cause autosomal recessive Stargardt disease in an American family. The two mutations cosegregated with the disease in the family with two affected siblings being compound heterozygotes, whereas eight other normal siblings and two parents carried only one or neither of the two mutations. The two mutations were absent in 200 normal controls. The family showed an uncommon *ABCA4*-related phenotype in which affected individuals present with classic juvenile Stargardt disease, but rapidly progress to show a severe cone-rod dystrophy clinical phenotype.

The novel compound heterozygous severe mutations may explain the distinct clinical features observed in the family. As proposed by Maugeri et al. [16], two null *ABCA4* alleles such as homozygous mutation IVS30+1G>T results in retinitis pigmentosa; compound heterozygosity for a null and a moderately severe mutation such as IVS30+1G>T and IVS40+5G>A causes cone-rod dystrophy; two moderately severe mutations or a mild and a severe allele such as IVS30+1G>T and 2588G>C (a mild mutation) cause Stargardt disease. In our study, the mutant c.655A>T allele in exon 6 causes early termination of the protein synthesis. The truncated protein has 218 residues, which is less than one-tenth of the wild-type protein. In either case, it will abolish a functional ABCR protein. In contrast, the c.5312+3A>T splicing mutation occurs after exon 37, which results in a mutant ABCR with the first 1,770 amino acid residues (80% of ABCR) intact and thus may cause a less severe effect on the function of ABCR. The combined effect of these two mutations thus may lead to the special clinical feature identified in the family under this study. Most recently Ben-Ya’acov et al. [17] studied 15 members of six consanguineous Arab families who reside in the same village and manifest a retinal degeneration that seems identical to the one we describe here. During early stages of disease, funduscopic and angiographic findings as well as retinal function resemble those of Stargardt disease, while later in life, severe, widespread cone-rod degeneration ensues. Marked progressive involvement of the retinal periphery distinguishes this phenotype from classic Stargardt disease. These authors found two novel *ABCA4* deletions, p.Cys1150del and c.4254–15del23. Interestingly, one of their patients who is a compound heterozygote for the deletions had typical Stargardt disease, while the remaining 14 patients, who were homozygous for the c.4254- 15del23 intronic deletion, had the progressive form of disease. The authors performed detailed RT–PCR analysis in normal retina and lymphoblastoid cells and could not find the normal full-length ABCA4 transcript in lymphoblastoid cells of affected homozygote patients who only showed alternatively spliced transcripts, indicating that homozygosity for the novel c.4254–15del23 splicing mutation is associated with a severe progressive form of disease. In summary, we characterized a family presenting a phenotype of progressive transition from Stargardt disease to severe cone-rod dystrophy. We identified two novel mutations in the ABCA4 gene as a causal for the disease in the family.

## Appendix 1. PCR primers for amplifying *ABCA4* exons

To access the data, click or select the words “Appendix 1.” This will initiate the download of a pdf archive that contains the file.
